# A survey of knowledge attitudes and practices relating to musculoskeletal examination amongst pediatricians in maharashtra, India

**DOI:** 10.1186/1546-0096-12-S1-P99

**Published:** 2014-09-17

**Authors:** Anita Dhanrajani, Nicola Smith, Helen Foster, Raju P Khubchandani

**Affiliations:** 1 Pediatrics, Jaslok Hospital and Research Center, Mumbai, India; 2 Musculoskeletal Research Group, Institute Cellular Medicine, Newcastle upon Tyne, UK

## Introduction

Musculoskeletal examination(MSKe) is a neglected clinical skill in paediatric practice and this could contribute to delayed or missed rheumatologic diagnosis in children. paediatric **G**ait **A**rms **L**egs **S**pine (pGALS), a simple format of MSKe, targeted at non specialists has been shown to be acceptable and valid. Its potential as a clinical tool in populations underserved by paediatric rheumatologists, such as India, has yet to be explored. This information could be used to design curricula and teaching methods to students and practitioners in such areas.

## Objectives

To assess the Knowledge, Attitude and Practices relating to MSKe in Paediatricians in one state of India.

## Methods

An internet based survey between March and April 2014 based on a published UK study of self reported confidence in MSKe in children [Jandial]. A questionnaire was emailed to all paediatricians on a professional database from Maharashtra state (capital-Mumbai).The questions (with Likert scale) related to profiling respondents qualifications / experience, clinical practice, MSKe training (in adults / children), confidence in MSKe compared to other systems, awareness / use of pGALS and free text comments regarding preferred technique of MSKe teaching. Responses were anonymised and three reminders were sent. This was deemed an audit of clinical practice without need for ethical approval.

## Results

The response rate was 223/1523 (14.64%). Most respondents, (180/223,80.72%) reported training in MSKe either in children (36.77%) or adults (6.28%) or both (37.67%). Despite this training 120/223, (53.81%) were confident in ‘some aspects’ and a lesser number (80/223,35.87%) in ‘most aspects’ of MSKe; notably respondents were less confident in MSKe compared to other systems. Respondents reported that physical exam comprised 1/3 of routine paediatric consultation, but MSKe was included in a minority (70/223, 31.39%) of routine consultations; many respondents (115/223, 51.57%) deemed MSKe integral to routine physical exam (albeit of these 62/115 ie 53.9% were not confident in their ability to perform MSKe) and the rest, performed MSKe only in the presence of overt MSK complaints. Notably of those who had been taught MSKe 58/180 (32.2%) actually performed this as a part of their routine consultation; of these respondents, 43.8% claimed to be confident in ‘most’ aspects. Most respondents (158/223,70.85%) were unaware of pGALS, although almost all (99%) expressed the need to include MSKe teaching in medical schools and 64% stated a need for training in both undergraduate and post graduate curricula. Of those unaware of pGALS, most (85%) expressed a desire to learn it and from a list of multiple options for format of resources, preferred ‘hands on’ workshops (50%), a CD demonstration (49.47%), or written materials (36.7%).

## Conclusion

This survey shows that at least for one state in India many paediatricians are not confident in MSKe and are less confident about it compared to other systems included in medical training. There is need for greater training and awareness about the importance of MSKe to those already in practice which should translate to its routine use with increased confidence. There is also a definite need to emphasize MSKe training in medical schools. Lastly as a simple validated skill, there is considerable potential to increase awareness about pGALS amongst paediatricians using easy methods and thereby improve performance of MSKe in clinical practice.

## Disclosure of interest

None declared.

